# P-1810. Development of Avian and Human Influenza Analytical Reference Materials for Diagnostics and Surveillance

**DOI:** 10.1093/ofid/ofaf695.1979

**Published:** 2026-01-11

**Authors:** Leka Papazisi, Holly Asbury, Jason Bose, Pushpa Gujjari, Laksmi Castro, Trudy Corriea, Loricel Champ, Brian Chase, Sung-Oui Suh, Joseph Thiriot, Kyle Young, Victoria Knight-Connoni

**Affiliations:** American Type Culture Collection (ATCC), Gaithersburg, MD; American Type Culture Collection (ATCC), Gaithersburg, MD; American Type Culture Collection (ATCC), Gaithersburg, MD; American Type Culture Collection (ATCC), Gaithersburg, MD; American Type Culture Collection (ATCC), Gaithersburg, MD; American Type Culture Collection (ATCC), Gaithersburg, MD; American Type Culture Collection (ATCC), Gaithersburg, MD; American Type Culture Collection (ATCC), Gaithersburg, MD; American Type Culture Collection (ATCC), Gaithersburg, MD; American Type Culture Collection (ATCC), Gaithersburg, MD; American Type Culture Collection (ATCC), Gaithersburg, MD; American Type Culture Collection (ATCC), Gaithersburg, MD

## Abstract

**Background:**

Human and highly pathogenic avian influenza (HPAI) viruses pose a major public health risk due to their potential for widespread illness and economic consequences. Early detection and control of outbreaks rely on effective surveillance and diagnostic testing.

**Figure 1:**
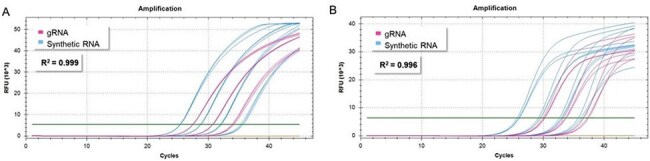
H5N1 qPCR data Figure 1: qPCR amplification curves generated with ATCC® VR-3436SD™ (subtype H5N1) (blue) and H5N1 gRNA (pink) using (A) a Hoffmann et al., 2016 assay targeting HA, and the (B) CDC Flu SC2 Multiplex assay targeting M.

**Figure 2:**
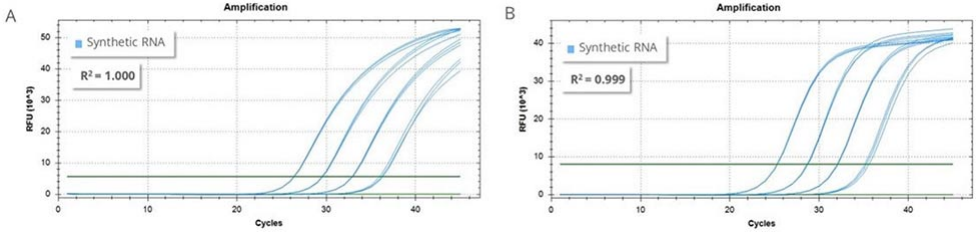
H5N6 qPCR data Figure 2: qPCR amplification curves generated with ATCC® VR-3439SD™ (subtype H5N6) using (A) a Hoffmann et al., 2016 assay targeting HA, and the (B) CDC Flu SC2 Multiplex assay targeting M.

**Methods:**

ATCC® developed a comprehensive suite of quantitative synthetic analytical reference materials (ARMs) for HPAI virus serotypes H5N1, H5N6, H7N7, H7N9, and H9N2; human influenza A virus serotypes H1N1, H3N2, and H1N1 2009 pandemic; and Influenza B virus strains. Each synthetic ARM contains the complete sequences from segments 4, 5, 6, 7, and 8, including the HA, NP, NA, M1, M2, NS1, and NEP/NS1 genes, covering 50% of the influenza genome. These segments are key diagnostic targets for molecular tests and provide sufficient genomic context for assessing assay specificity. These ARMs are manufactured using a highly reliable synthetic biology technology, verified through next-generation sequencing, and quantitated via Droplet Digital PCR (Bio-Rad Laboratories, Inc). Further, they do not contain any viable material and can be handled in BSL-1 settings. As such, they are intended to serve as safe and reliable positive controls for molecular tests for surveillance and diagnostics.

**Figure 3:**
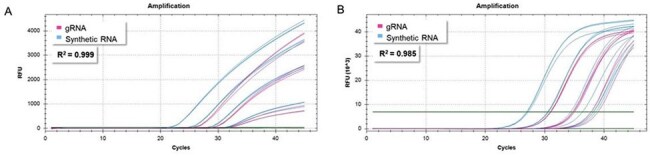
H9N2 qPCR data Figure 3: qPCR amplification curves generated with ATCC® VR-3440SD™ (subtype H9N2) using (A) a Hassan et al., 2022 assay targeting HA, and the (B) CDC Flu SC2 Multiplex assay targeting M.

**Figure 4:**
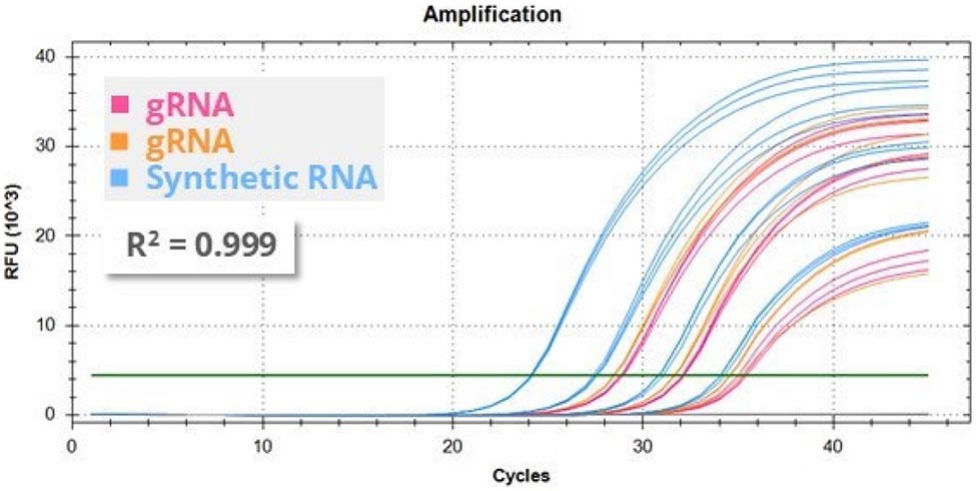
pdm09 qPCR data Figure 4: qPCR amplification curves generated with synthetic RNA ATCC® VR-3388SD™ (H1N1 pdm09) and genomic RNA samples derived from two viral cultures ATCC® VR-3441 ™ VR-1988™, which are also both of H1N1 pdm09 type, using the WHO assay for the detection of Influenza type A subtype H1pdm09.

**Results:**

The synthetic ARMs were experimentally evaluated using several published quantitative PCR assays, including those from the Centers for Disease Control and Prevention, the World Health Organization, the World Organization for Animal Health, and other highly cited sources. We also conducted an in silico assessment of ARM compatibility with over 250 publicly available published assays. The synthetic products displayed equal performance to genomic RNA during all experimental tests.

**Conclusion:**

The data demonstrate that all synthetic ARMs for avian and human influenza are effectively designed and suitable for developing and validating molecular-based detection and quantification assays. Furthermore, our findings indicate that the synthetic RNA ARMs are equivalent to their corresponding genomic RNA, making them a valuable BSL-1 alternative to BSL–3–derived materials. Our results suggest that these synthetic ARMs can serve as reliable and safe controls for molecular assays used in diagnostics and surveillance.

**Disclosures:**

All Authors: No reported disclosures

